# Improvement of Multifactorial Tremor in Klinefelter Syndrome With Testosterone Replacement Therapy

**DOI:** 10.1155/crie/8988814

**Published:** 2026-08-20

**Authors:** Jared Houssarini, Albert Hsieh, Samantha Hocking

**Affiliations:** ^1^ Central Clinical School, University of Sydney, Sydney, Australia, sydney.edu.au; ^2^ Royal Prince Alfred Hospital, Sydney, Australia, nsw.gov.au; ^3^ Boden Initiative, Charles Perkins Centre, University of Sydney, Sydney, Australia, sydney.edu.au

## Abstract

A man in his 50s with long‐standing learning difficulties and schizophrenia currently treated with clozapine presented with gynaecomastia and a new right testicular mass. He was noted to have a severe tremor interfering with activities of daily living treated with combination levodopa + benserazide, primidone and topiramate. Investigations revealed elevated prolactin, gonadotropins, sex hormone binding globulin, total testosterone and oestradiol. Right orchidectomy revealed Leydig cell hyperplasia. Karyotyping diagnosed Klinefelter syndrome. Testosterone replacement therapy (TRT) was commenced, with subsequent marked improvement in his debilitating tremor. Tremor is a potentially disabling complication of Klinefelter syndrome with fewer than 50 cases reported in the literature. The pathophysiology of tremor in Klinefelter syndrome is unclear. Tremor in Klinefelter syndrome is often refractory to anti‐tremor medical therapy; however, as reported in this case, testosterone therapy may improve tremor in Klinefelter syndrome. It is rare for TRT to significantly improve tremor as reported in this case.

## 1. Introduction

Klinefelter Syndrome is a genetic disorder characterised by the presence of additional X chromosomes in males (designated as 47, XXY). Klinefelter syndrome is the most common cause of male infertility, affecting 1 in 500–600 men [1]. It is classically associated with hypergonadotropic hypogonadism, infertility and a variety of physical and cognitive impairments, such as low muscle tone (resulting in impairments of fine and gross motor skills), gynoid body fat distribution, speech, language and learning difficulties and neuropsychiatric conditions such as autism, anxiety, ADHD, depression and schizophrenia [2–5]. Among the less commonly discussed manifestations of Klinefelter syndrome are movement disorders, including tremors, which are overrepresented compared to the general population [6], with the prevalence of tremor in Klinefelter syndrome being up to 63% in some case series [7, 8]. Despite this, the literature is sparse regarding tremor, with fewer than 50 previously published case reports addressing tremor and its management in Klinefelter syndrome. Of these, several case series have reported the effects of testosterone replacement therapy (TRT) on tremor in eight men with Klinefelter syndrome: two with benefit, five with a neutral effect, and one with a negative effect [8–11]. This case study adds to the literature supporting the possible beneficial effect of TRT in patients with Klinefelter Syndrome and tremor, though the mechanism remains unclear and unexpected.

## 2. Case Presentation

A man in his late 50s with long‐standing schizophrenia was referred for evaluation of gynaecomastia, elevated prolactin (five times upper limit of reference range) and a new right testicular mass. His medical history included chronic paranoid schizophrenia treated with clozapine, glaucoma and mixed anxiety and depression disorder. He reported excellent adherence to his medications, including his regular antipsychotics. He reported struggling with learning difficulties at school and was diagnosed with dyslexia as a child. He reported not drinking alcohol, and there was no history of previous significant alcohol intake. He had no history of any recreational substance or stimulant use. There was no history of anabolic steroid use. He lived alone in social housing in regional Australia.

On presentation, he was noted to have a long‐standing severe tremor treated with combination levodopa + benserazide, primidone and topiramate. The tremor affected his hands bilaterally, worse on his non‐dominant hand but still functionally impactful on his dominant hand. The tremor caused significant interference with his activities of daily living. Tasks, such as household duties, dressing and food preparation that require fine motor skills, were reported as difficult or impossible due to his tremor, requiring help from carers. He specifically reported being unable to drink from a cup, with both hands, instead needing to drink from a closed vessel or straw.

On examination, he presented with a dystonic posture of his head and neck with a slight head tremor. He was of average height at 162 cm and weighed 68 kg with a BMI of 26 kg/m^2^, with palpable gynaecomastia, moderate beard growth, low muscle bulk and a gynoid distribution of body fat with palpable bilateral gynaecomastia and bilateral small testes (~3 mL). There was a slight rest tremor in both hands and moderate postural and action tremors in both hands, worse on the left, around 2–3 cm in amplitude. There were no Parkinsonian signs in his upper or lower limbs, with no rigidity or bradykinesia being noted. The gait was normal. Hand tremor was most noticeable when attempting to drink from a cup or write with a pen.

The hormone profile found elevated gonadotropins, total testosterone, oestradiol, DHEA, androstenedione, 17‐OH progesterone, prolactin and sex‐hormone binding globulin (SHBG) (Table 1). His ACTH‐cortisol axis, thyroid function and IGF‐1 level were within normal reference limits. Testicular ultrasound revealed bilateral small testes (2.1 mL right and 2.7 mL left) with a 3‐mm ovoid isoechoic, well‐circumscribed lesion on the right testicle. MRI of the brain reported no sellar or suprasellar lesions.

**Table 1:** Laboratory evaluation at baseline and post‐orchidectomy.

Parameters	Baseline	Post‐orchidectomy	Reference range	Units
Total testosterone	32.1	21.8	9.5–28.0	nmol/L
Sex hormone binding globulin (SHBG)	162	190	15–50	nmol/L
Free androgen index (FAI)	19.8	11.5	15–100	%
Calculated free testosterone	205	113	130–570	pmol/L
Prolactin	2594	540	85–500	mIU/L
FSH	27.2	54.3	1–12	IU/L
LH (Abbott method)	22.2	41.8	0.6–12	IU/L

He subsequently underwent a right‐sided orchidectomy to investigate his testicular mass. Histopathology of his right testes revealed testicular atrophy with Leydig cell hyperplasia, confirming benign disease. Note that our endocrinology service was only consulted after the right orchidectomy had already been planned by the first treating team to exclude malignancy.

Repeat biochemical investigations post‐orchidectomy reflected an ongoing elevation in SHBG, prolactin, oestradiol and gonadotropins, with total testosterone within reference limits but low calculated free testosterone (Table 1). Elevated prolactin was consistent with antipsychotic therapy. The SHBG elevation was attributed to concurrent anti‐seizure medication (for the management of tremor) [12] and elevated oestradiol level [13] and was masking the presence of hypogonadism due to the normal range of total testosterone. Karyotyping confirmed karyotype 47, XXY. There was no mosaicism. Bone mineral density scan revealed osteopenia at the lumbar spine (T‐score L1–L4, −2.4) and osteoporosis at the total hip (T‐score, −2.7).

He was commenced on transdermal testosterone gel 12.5 mg once daily with dose titration to 37.5 mg daily over the subsequent months. At the time of writing, his dose was still being uptitrated.

His tremor was noted to markedly improve on commencing testosterone replacement, although not eradicated. The patient reported that he could now be more independent in his activities of daily living, including preparing food and drinking from open vessels. No changes to his other medications, including combination levodopa + benserazide, fluoxetine, clozapine, primidone or topiramate, were made during this time (Table 2).

**Table 2:** Case summary.

Patient demographics and phenotype	Caucasian male Age in late 50s Height 162 cm, weight 68 kg, BMI 26 kg (m)^2^ Living alone in regional Australia
Background	Chronic schizophrenia, anxiety/depression, psoriasis Severe dystonic tremor, dyslexia, childhood learning difficulties, glaucoma, bilateral cataracts, vitamin D deficiency, SCC/BCC, childhood asthma
Medications	Clozapine 25 mg mane, 375 mg nocte Fluoxetine 20 mg mane Levodopa + benserazide 200/50 mg TDS Primidone 250 mg QID Topiramate 100 mg BD Latanoprost 125 μg OU nocte
Examination	Palpable gynaecomastia Moderate beard growth Low muscle bulk Gynoid distribution body fat Bilateral small testes Postural action tremor, worse on left, 2–3 cm amplitude
Investigations	Elevated SHBG, prolactin, oestradiol and gonadotropins Normal total testosterone with low free testosterone Karyotype 47, XXY without mosaicism

## 3. Discussion

The pathophysiology of tremor in Klinefelter syndrome remains poorly understood [6]. While there may be a degree of cerebellar volume loss and corpus callosum under development contributing to motor control issues and tremors in patients with Klinefelter syndrome [14], it remains likely that the aetiology of the tremor is multi‐factorial. Harlow and Gonzalez‐Alegre [7] postulate that excess activation of X chromosome genes may cause tremor by altering neurodevelopment or function, acting independently of genes located on somatic chromosomes responsible for essential tremor. It has previously been noted that men with Klinefelter syndrome have an increased prevalence of tremor, with prevalence rates of up to 63% in Klinefelter syndrome compared to 14% in controls [7].

Regarding the mechanism of action for TRT improving tremor, a 2005 animal study by Huppenbauer et al. [15] found that androgens enhance functional regeneration and recovery of motor neurones, and some studies suggest that TRT may improve motor coordination and reduce neurological soft signs in patients with Klinefelter syndrome [16]. Similar to our case, previous case reports (Table 3) have noted an improvement in tremor in patients with Klinefelter syndrome after the commencement of TRT. A case study in 2009 of a 71‐year‐old man with Klinefelter syndrome reported improved tremor [11] after administering monthly intramuscular TRT for only 3 months, resulting in rapid improvement in handwriting and resting tremor. Similarly, a 27‐year‐old man with Klinefelter syndrome demonstrated improvement of myoclonus and essential tremor with TRT [9]. In contrast, a case report by Harlow and Gonzalez‐Alegre [7] showed no improvement in tremor after TRT, and a case study in 2014 showed marked worsening in tremor amplitude after 3 months of weekly testosterone 200 mg intramuscular injections [10]. This variability suggests that while some patients with Klinefelter syndrome may experience symptomatic relief from TRT, others might encounter adverse effects on tremor, muddying the therapeutic approach.

**Table 3:** Previously published effects of testosterone on tremor in Klinefelter syndrome.

Literature	Year published	Karyotype	Tremor: age at onset	Tremor response to testosterone therapy
Harlow and Gonzalez‐Alegre [7]	2008	47, XXY	63	No effect
Kinoshita et al. [11]	2009	47, XXY	71	Improvement
Koegl‐Wallner et al. [8]	2014	47, XXY	10	No effect
		47, XXY	3	No effect
		47, XXY	Childhood	No effect
Burdick et al. [10]	2014	47, XXY	13	Worsened
Buijink et al. [9]	2014	47, XXY	Childhood	Improvement
Rabin et al. [6]	2015	47, XXY	N/A	No effect

Regardless, TRT in Klinefelter’s syndrome should not be based upon testosterone levels alone as there is often Leydig cell dysfunction and heterozygosity in patient virilisation phenotypes. Total testosterone may be normal or even elevated when SHBG is raised, as in our patient, in whom SHBG was further increased by elevated oestradiol and concurrent anti‐seizure medication; gonadotropins, calculated free testosterone, and the free androgen index give a more reliable estimate of androgen status in this setting. In our case, removal of the testicular tissue was associated with a fall in total and calculated free testosterone and a rise in gonadotropins (Table 1), unmasking overt biochemical hypogonadism that informed the subsequent decision to commence testosterone replacement.

Sex hormone‐binding globulin is often elevated in patients with Kinefelter’s syndrome [17], and in our patient, the above‐range total testosterone was attributed to markedly elevated SHBG, driven by elevated oestradiol and concurrent anti‐seizure therapy; the concurrently low‐normal and subsequently low calculated free testosterone and FAI indicate that the elevated total testosterone likely reflected SHBG binding rather than true androgen excess. TRT is integral in treating patients with Klinefelter’s syndrome for positive changes in mood and behaviour, muscle mass and bone integrity [17], as demonstrated in our patient.

This case highlights the challenge of recognising the diagnosis of Klinefelter syndrome even in the presence of severe debilitating comorbidities such as dyslexia, schizophrenia and tremor. Unfortunately, this is not uncommon with Klinefelter syndrome, in particular if not presenting with a tall stature and very clear signs of hypogonadism, as was the case with our patient. In many patients with Klinefelter syndrome, testosterone levels may be in the normal or low‐normal range [18]. This case study adds to the literature supporting the possible beneficial effect of testosterone in patients with Klinefelter syndrome and tremor, though the mechanism remains unclear and unexpected. Establishing whether a dose–response relationship between testosterone and tremor exists may require prospectively documented cases or registry data in the future.

## Funding

Open access publishing facilitated by The University of Sydney, as part of the Wiley—The University of Sydney agreement via the Council of Australasian University Librarians.

## Conflicts of Interest

The authors declare no conflicts of interest.

## Data Availability

The data that support the findings of this study are available from the corresponding author upon reasonable request.
